# Targeted Delivery of GDNF through the Blood–Brain Barrier by MRI-Guided Focused Ultrasound

**DOI:** 10.1371/journal.pone.0052925

**Published:** 2012-12-27

**Authors:** Feng Wang, Yu Shi, Lin Lu, Li Liu, Youli Cai, Hairong Zheng, Xin Liu, Fei Yan, Chao Zou, Chengyu Sun, Jie Shi, Shukun Lu, Yun Chen

**Affiliations:** 1 Department of Ultrasound, Peking University Shenzhen Hospital, Biomedical Research Institute, Shenzhen PKU-HKUST Medical Center, Shenzhen, China; 2 National Institute on Drug Dependence, Peking University, Beijing, China; 3 Paul C. Lauterbur Research Center for Biomedical Imaging, Institute of Biomedical and Health Engineering, Shenzhen Institutes of Advanced Technology, Chinese Academy of Sciences, Shenzhen, China; 4 Department of Physiology and Neurobiology, Xinxiang Medical University, Xinxiang, China; 5 Shenzhen Key Laboratory for MRI, Shenzhen, China; St. Jude Children's Research Hospital, United States of America

## Abstract

Neurotrophic factors, such as glial cell line-derived neurotrophic factor (GDNF), are promising therapeutic agents for neurodegenerative diseases. However, the application of GDNF to treat these diseases effectively is limited because the blood–brain barrier (BBB) prevents the local delivery of macromolecular therapeutic agents from entering the central nervous system (CNS). Focused ultrasound combined with microbubbles (MBs) using appropriate parameters has been previously demonstrated to be able to open the BBB locally and noninvasively. This study investigated the targeted delivery of GDNF MBs through the BBB by magnetic resonance imaging (MRI)-guided focused ultrasound. Evans Blue extravasation and histological examination were used to determine the optimum focused ultrasound parameters. Enzyme-linked immunosorbent assay was performed to verify the effects of GDNF bound on MBs using a biotin–avidin bridging chemistry method to promote GDNF delivery into the brain. The results showed that GDNF can be delivered locally and noninvasively into the CNS through the BBB using MRI-guided focused ultrasound combined with MBs under optimum parameters. MBs that bind GDNF combined with MRI-guided focused ultrasound may be an effective way of delivering neurotrophic factors directly into the CNS. The method described herein provides a potential means of treating patients with CNS diseases.

## Introduction

Neurotrophic factors have emerged as promising tools for the treatment of various neurodegenerative diseases. The strong trophic effect of glial cell line-derived neurotrophic factor (GDNF) on the dopaminergic system makes it one of the most potent neurotrophic factors for the treatment of Parkinson's disease (PD) [1]. GDNF is also an essential growth factor for the development of the kidneys and spinal cord motoneurons, and it exerts a wide range of effects on peripheral and central neurons [2]. Several studies have suggested that GDNF is a potential target in the treatment of drug addiction. Over the past several years, accumulating evidence has shown that GDNF plays a regulatory role in drug abuse, including psychostimulants, morphine, and alcohol [3], [4], [5]. Although increased GDNF levels in the central nervous system (CNS) may be beneficial to the treatment of neurodegenerative diseases, such as PD and drug addiction [5], [6], the therapeutic application of GDNF is limited because efficient methods of delivering it to the CNS are currently not available.

The CNS is protected from the entry of foreign substances by a barrier system known as the blood–brain barrier (BBB). However, this very protective barrier for the brain also blocks most therapeutic agents from entering the brain parenchyma from the circulation, thus hampering treatment of CNS diseases [7], [8]. GDNF has a molecular size of 24 kDa and is easily blocked by the BBB. Its delivery to the CNS requires the temporary opening of the BBB to allow larger molecules to penetrate it. Thus, the development of safe and efficient techniques to deliver therapeutic agents across the BBB into the brain interstitium is critical for the treatment of CNS diseases.

Ultrasound imaging techniques can serve as diagnostic and therapeutic tools. Experimental evidence suggests that focused ultrasound selectively disrupts the BBB and that its combination with magnetic resonance imaging (MRI) permits image-guided target planning and real-time temperature mapping during tumor sonication [9]. Ultrasound combined with ultrasound contrast agents opens the BBB safely and reversibly under certain ultrasonic parameters, such as pressure amplitude, repetitive frequency, exposure time, and delay time, among others [10]. Many studies have reported the effects of the resonant frequency of the transducer, microbubble (MB) dosage, and peak negative pressure on BBB permeability [10], [11], [12], and some others have demonstrated success in injecting therapeutic or diagnostic agents followed by MBs into the body [10], [13]. However, the integrated effects of these parameters on BBB permeability as well as a comparison of the effects of intravenous injection of therapeutic agents followed by MBs and attachment of agents to the surface of MBs using a biotin–avidin bridging system on therapeutic agent delivery into brain tissue have not been reported to date.

The purpose of this study was to explore (1) the optimum parameters for disrupting the BBB using an orthogonal design and (2) the feasibility of delivering GDNF through the BBB using an MRI-guided focused ultrasound BBB disruption method. Two methods of protein delivery, namely, GDNF bound on MBs and GDNF injected with MBs, were compared to determine the best way of promoting GDNF delivery and the best biological route for material transport into the brain.

## Materials and Methods

### Animal Preparation

All procedures in the animal experiments were conducted in accordance with the guidelines developed by the National Institutes of Health and approved by the Institutional Animal Care and Use Committee of Peking University Shenzhen Hospital (Permit No. 09-215). Male Sprague–Dawley rats weighing between 280 and 350 g were used in this study. Before focused ultrasound sonication, the animals were intraperitoneally anesthetized with chloral hydrate (300 mg/kg). Hair on the skull was removed, and each animal was placed in the supine position on a sonication table.

### Ultrasound Equipment

The system used to generate ultrasound energy in all the experiments comprised a function generator (AGF3022B; Tektronix, USA), an RF amplifier (DC2500A; AR, Souderton PA, USA), and a custom-made passive L–C matching circuit. Ultrasound waves were generated using a single-element focused ultrasound transducer (Valpey Fisher, Hopkinton, MA, USA). The diameter of the ultrasound transducer was 100 mm, and the radius of the curvature was 50 mm. The transducer was attached to a three-axis motorized positioning system to allow fine positioning of the focal volume. Three transducers with corresponding resonant frequencies of 0.8, 1.0, and 1.2 MHz were used. Each transducer was calibrated using a hydrophone. Peak negative pressure amplitude levels were kept constant over the duration of each sonication at 0.6, 0.8, and 1.1 MPa (peak negative pressure amplitude calibrated by the hydrophone in water) [14]. Sonication was pulsed at a burst length of 10 ms and repetition frequency of 1 Hz (duty cycle, 1%). Total sonication times of 30, 60, and 90 s were used.

### Choice of Optimum Parameters based on an Orthogonal Design

An L_18_ (3^7^) orthogonal design was used (Table 1). Each group was composed of six rats, and each rat was analyzed thrice to obtain the average OD value of Evans Blue (EB) in the brain. Five factors, namely, transducer frequency, MB dosage, exposure time, peak negative pressure, and delay time, were designed for the orthogonal test with three levels, for a total of 18 treatments. EB extravasation was used as the index for the optimum parameters of BBB opening. As the degree of tissue damage must be considered, hematoxylin–eosin (HE) staining, TUNEL staining, and electron microscopy were used to further confirm the optimum parameters.

**Table 1:** L_18_ ( 3^7^ ) orthogonal expermiental design.

Level	transducer frequency	microbubble dosage	exposure time#	peak negative-pressure*	delay time$
	(MHz)	(ml)	(s)	(MPa)	(s)
1	0.8	0.2	30	0.6	15
2	0.8	1.0	60	0.6	120
3	0.8	0.2	90	1.1	60
4	0.8	0.5	30	1.1	15
5	0.8	0.5	60	0.8	120
6	0.8	1.0	90	0.8	60
7	1.0	0.5	30	0.8	60
8	1.0	0.2	60	1.1	15
9	1.0	1.0	90	0.6	120
10	1.0	1.0	30	0.6	15
11	1.0	0.5	60	0.8	60
12	1.0	0.2	90	1.1	120
13	1.2	1.0	30	1.1	120
14	1.2	0.5	60	1.1	60
15	1.2	1.0	90	0.8	15
16	1.2	0.2	30	0.8	120
17	1.2	0.2	60	0.6	60
18	1.2	0.5	90	0.6	15

(#) Ultrasonic irradiation time.

(*) The Negative peak value of acoustic pressure measured by hydrophone in water.

($) The time from injecting microbubbles into vessels to ultrasonic irradiation.

### Assessment of BBB Integrity

BBB permeability was evaluated by EB extravasation into brain tissue. EB (2%, 4 ml/kg) was injected through the tail vein of anaesthetized rats 1 min after sonication. After 4 h, six rats in each group were anesthetized and perfused with phosphate-buffered saline (PBS). The dyed brain tissues were immediately dissected and removed. Samples were weighed and then incubated in a 10× volume of formamide (Sigma-Aldrich, St. Louis, MO, USA) at 60°C for 72 h. The concentration of EB was then determined spectrophotometrically at 620 nm. Sample value calculations were based on EB dye standards mixed with the same solvent. Results were expressed as mean±standard error of the mean (SEM).

### Tissue Preparation and Histological Examination

All rats were sacrificed approximately 4 h after sonication for histological evaluation. Their brains were immediately removed and fixed in 10% buffered neutral formalin. The blue-stained focal region was embedded in paraffin. A series of sections parallel with the beam direction across the MRI slices were cut for HE staining. TUNEL staining was used to detect apoptotic cells in the neighboring sections of the focal plane. The sections were counterstained with hematoxylin.

Electron microscopy was selected to evaluate the ultrastructural basis of BBB disruption. Five rats per group were anesthetized as described above. Their brains were fixed by perfusion using 2.5% paraformaldehyde and 1.5% glutaraldehyde in 0.1 M phosphate buffer (pH 7.2). Sections measuring approximately 0.5 mm^3^ from the sonicated (blue spots) and unsonicated (controls) areas were fixed for 2 h in the same fixative and then treated for electron microscopic observation.

### GDNF–MB Conjugation

The MBs used in this study were biotinylated and lipid coated, and they encapsulated a high-molecular-weight gas core of perfluoropropane (C3F8) [13]. The MBs were conjugated to GDNF by means of a multistep biotin–avidin bridging chemistry method as described previously but with some modifications [15]. Briefly, the biotinylated MBs were incubated with streptavidin in PBS for 60 min at 4°C, and the unbound streptavidin was removed by static flotation. The streptavidin-coated biotinylated MBs were then incubated with biotinylated GDNF for 60 min at 4°C, and the unbound GDNF was removed by static flotation by washing with PBS thrice. The MBs were thus prepared with biotinylated GDNF coupled to the phospholipid monolayer of the MB shell through a biotin–streptavidin bridge. The MB radius was measured using an Accusizer (Model 780A; Particle Sizing System, Santa Barbara, CA, USA) at a typical concentration of 10^9^ MB/ml, yielding a mean radius of 2.5 µm.

### Determination of Adhesion Efficiency

An indirect method was used to determine adhesion efficiency. Briefly, after centrifugation of the MBs at 400×*g* for 3 min, the supernatant was collected and the concentration of GDNF in the samples was determined by enzyme-linked immunosorbent assay (ELISA). Adhesion efficiency was defined as follows: Encapsulation Efficiency (%) = Amount of Drug Bound on MBs (µg)/Initial Amount of Drug (µg)×100. The amount of drug bound on MBs was determined by subtracting the amount of free drug in the supernatant from the total. Results were reported as mean±SD (*n* = 3).

### In Vitro Bioactivity Assay

The bioactivity of GDNF bound on the MBs was assessed using a PC-12 cell line that was purchased from American Type Culture Collection (Rockville, MD, USA). A previous study showed that GDNF is able to induce neurite outgrowth in PC-12 cells [16]. PC-12 cells were plated onto a 12-well culture plate at a low density of 2×10^3^ cells/cm^2^. MBs that bound GDNF (MB-GDNF) (50 ng/ml GDNF content) or purified GDNF (50 ng/ml) were added 24 h later, and neurite outgrowth was visualized under an inverted microscope after 7 days in culture.

### Sonication

The focused ultrasound system was placed inside a 3.0 T MRI scanner that was used to image the brain and target the ultrasound beam. T1-weighted images were obtained to aid in the selection of target locations in the brain. After injecting GDNF (3 mg/kg) or a 0.5 ml bolus of MB-GDNF (3 mg/kg GDNF content) into the tail vein (*n* = 6), sonication was performed. A 0.5 ml bolus of an MB-based ultrasound contrast agent, in which the agent contained 5–8×10^8^/ml, with a mean size of 2–5 µm, was injected simultaneously in the GDNF group. The brain of each rat was sonicated from the dorsal surface into the right hemisphere at a depth of 2–3 mm. As the rat brain measures 5–6 mm along the axis of the ultrasound beam path, the skull base was also sonicated during the procedure. After the sonication procedure and acquisition of MR images were completed, EB (100 mg/kg; Sigma, St. Louis, MO, USA) was injected through the tail vein to mark and confirm the site of BBB disruption on tissue blocks. The unsonicated group served as the control for the integrity of the undisrupted BBB.

### MRI

The MRI scanner used was a standard 3.0 T Signa system (TRIO 3.0 T MRI; Siemens MAGNETOM, Erlangen, Germany). Anatomical images were acquired in multiple planes prior to and after sonication using a T2-weighted fast spin echo sequence (TE = 16 ms; TR = 1000 ms; ETL = 4; BW = 16 kHz; matrix, 184×256; slice = 1 mm; NEX = 2; FOV = 5 cm) to evaluate whether signs of tissue damage were present after exposure. The rats were anesthetized with 30% chloral hydrate during the imaging procedure. A 7.5 cm diameter surface coil was placed under the head. Sonications were performed through a hole in the coil that was filled with a bag containing degassed water. A gradient echo sequence was used to aim the beam at the brain. Following each sonication, T2-weighted fast spin echo images were obtained and repeated after an intravenous bolus injection of meglumine gadopentetate MR contrast agent (0.1 ml/kg; Consun, Guangzhou, China) to detect and evaluate the opening of the BBB.

### GDNF Detection and Quantification

The rats were sacrificed 4 h after sonication, and their brains were removed immediately afterward (*n* = 6). Sonication spots, easily detected by EB staining, were harvested. The preparation of brain homogenates for the GDNF assay was conducted as described previously [17]. Briefly, the brains were dissected and homogenized in a modified radioimmunoprecipitation assay buffer. The homogenate was centrifuged at 14,000×*g* for 30 min at 4°C, and the supernatant was collected. GDNF in the tissue was quantified using a GDNF ELISA kit (R&D Systems, Minneapolis, MN, USA) according to the manufacturer's protocol.

### Statistical Analysis

Data were expressed as mean±SEM. Analysis of the orthogonal design results was performed using SPSS 13.0. Comparisons between groups were analyzed using one-way ANOVA. *p*<0.05 was considered statistically significant.

## Results

### Determination of Adhesion Efficiency

The concentration of GDNF in the supernatant was determined by ELISA. The results were obtained using the formula mentioned above. The adhesion efficiency of MBs was 63.1%±3.5%.

### Choice of Optimum Parameters

#### Analysis of orthogonal experiment

**Figure 1:**
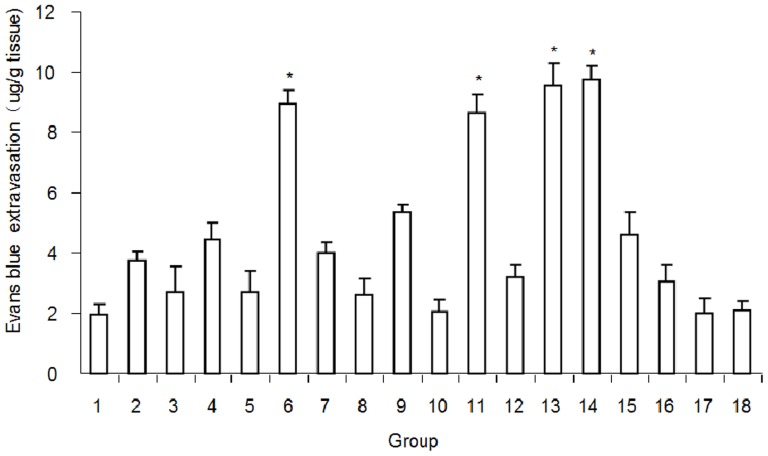
Evans Blue extravasation under the ultrasonic conditions presented in **Table 1**. Evans Blue extravasation (mean ± SEM) was significantly higher in Groups 6, 11, 13, and 14 than in the other groups. *p<0.01, compared with the other groups besides groups 6, 11, 13 and 14.

After ultrasound according to the conditions presented in Table 1, BBB disruption, quantified by EB extravasation, was measured in the targeted brain region. The numbers from spectrophotometer analysis showed that EB extravasation within the sonicated brain was significantly higher in Groups 6, 11, 13, and 14 than in the other groups (*p*<0.01; Fig. 1). The effects of various factors on EB extravasation were analyzed by ANOVA and could be ranked as follows: pressure amplitude>repetitive frequency>exposure time>MB dosage>delay time. The delay time had no significant effect on the experimental results (*p*>0.05). EB extravasation was not observed in the control group (unsonicated group). The EB concentration calculated from the OD value was 0 and thus is not shown in the diagram. Histological examination, including HE staining, TUNEL staining, and electron microscopy, was used to further analyze the experimental results.

#### Histological evaluation after focused ultrasound-induced BBB disruption in rats

**Figure 2:**
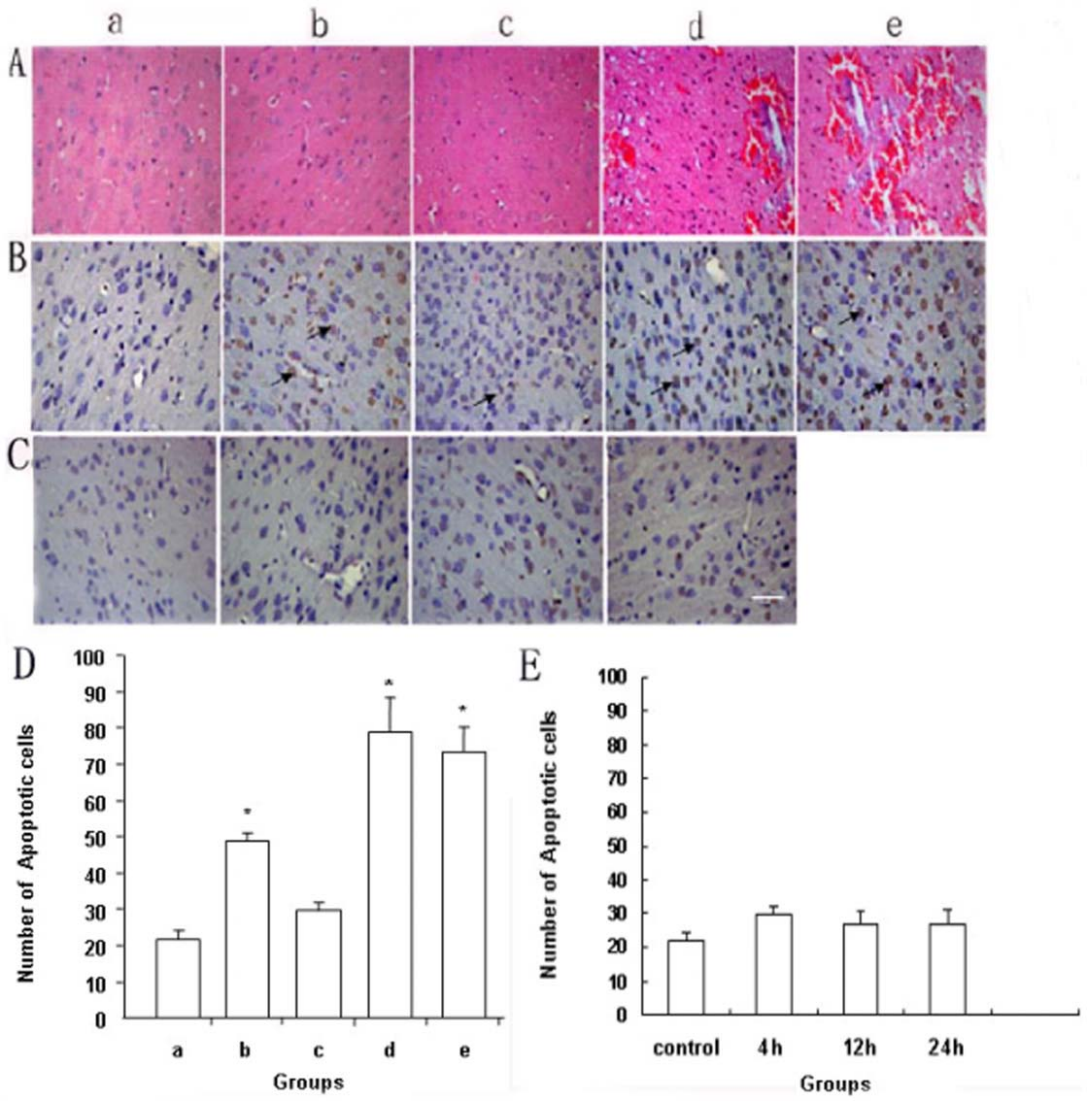
Histological examination and analysis of harvested brain tissue showing BBB disruption induced by focused ultrasound. (A) HE staining of brain sections from the sonicated target. The targeted tissues in Groups 13 and 14 exhibited various pathological changes, such as tissue necrosis and erythrocyte exudation. (B) TUNEL staining was used to identify apoptotic cells in brain sections from the sonicated target (arrows): a, Control group (unsonicated group); b, Group 6; c, Group 11; d, Group 13; e, Group 14 in panels A, B, and D. (C) TUNEL staining of harvested brain tissue for 4, 12, and 24 h after BBB disruption under the ultrasonic conditions in Group 11: a, Control group (unsonicated group); b, 4 h after BBB disruption; c, 12 h after BBB disruption; d, 24 h after BBB disruption in panels C and E. (D) Quantification of tissue damage. The number of apoptotic cells in each specimen was counted in five separate experiments. Values are expressed as mean±SEM. *p<0.01, compared with the control group and Group 6. (E) The number of apoptotic cells in each specimen was counted in five separate experiments. Values are expressed as mean±SEM. Significant differences between groups were not detected. The scale bars in panels A–C represent 50 µm.

The damage caused by focused ultrasound-induced BBB disruption was carefully assessed. When BBB disruption occurred under the ultrasonic conditions based on the parameters in Groups 13 and 14, a few scattered extravasated red blood cells were observed (Fig. 2A-d and A-e). Severe damage was not detected in the other groups. Some TUNEL-positive apoptotic cells were observed at the sites with the most severe extravasation in Groups 6, 13, and 14. The number of TUNEL-positive apoptotic cells was greater in these groups than in Group 11 (Fig. 2B and D), which can be attributed to the ultrasound under different parameters. TUNEL staining of harvested brain tissue was detected for 4, 12, and 24 h after BBB disruption under the ultrasonic conditions in Group 11 to observe whether there was an increasing trend of apoptosis with time under the same parameters (Fig. 2C). The results showed that there was no significant difference between groups (*p*>0.05; Fig. 2E).

#### Electron microscopic observation of BBB disruption

**Figure 3:**
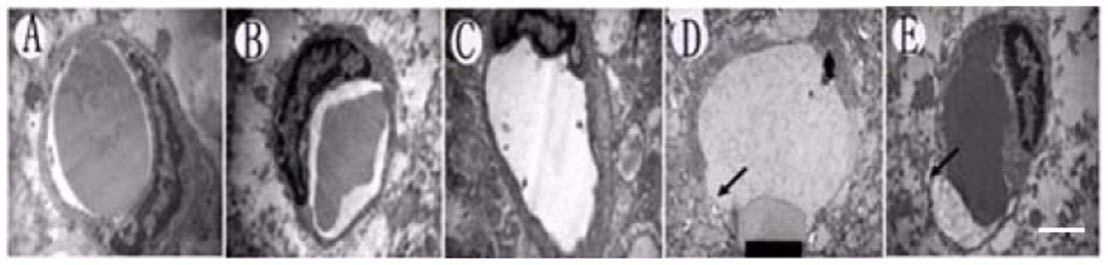
Ultrastructural electron microscopic observation of cell damage. (A) Control group (unsonicated group). (B) Group 6. (C) Group 11. (D) Group 13. (E) Group 14. Endothelial and nerve cells were severely damaged in Groups D and E, but ultrastructural changes were not observed in Groups A–C. The scale bar represents 150 nm.

For the animals sacrificed approximately 4 h after sonication, tight junctions in some of the vessels appeared to open, indicating that endothelial cells were severely damaged in Groups 13 and 14 (Fig. 3D and E). The endothelial cells appeared to retain their integrity and were not obviously damaged in Groups 6 and 11(Fig. 3B and C).

### Bioactivity of GDNF Bound on MBs

**Figure 4:**
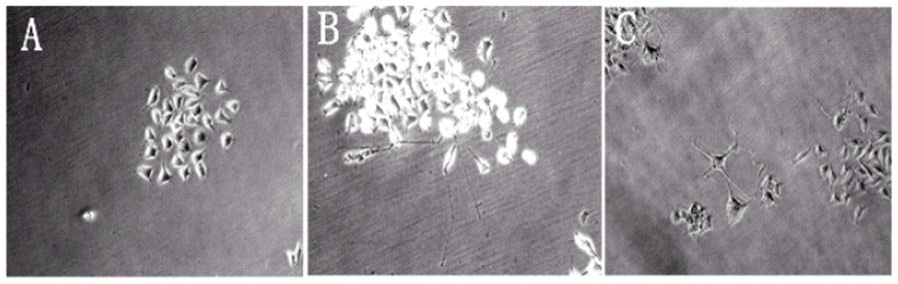
Differentiation of PC-12 cells cultured with MB-GDNF. (A) MBs without GDNF. (B) MB-GDNF. (C) Purified rat recombinant GDNF.

PC-12 cells derived from rat pheochromocytoma presented an undifferentiated aspect when grown in culture. When bioactive GDNF was added to the culture medium, the cells differentiated and began to develop a neural phenotype visualized by the presence of neuronal-like processes. As shown in Fig. 4A, no neurite outgrowth was observed in the cells treated with MBs without GDNF. In contrast, GDNF bound on MBs was able to differentiate PC-12 cells after 7 days of treatment, indicating that the bound neurotrophic factor remained biologically active after the biotinylation process (Fig. 4B). A similar differentiation was observed in the cells treated with the same amount of purified rat recombinant GDNF (Fig. 4C). These results demonstrated that biotinylated GDNF bound on MBs was biologically bioactive.

### BBB Disruption Induced by MRI-guided Focused Ultrasound in Rats

**Figure 5:**
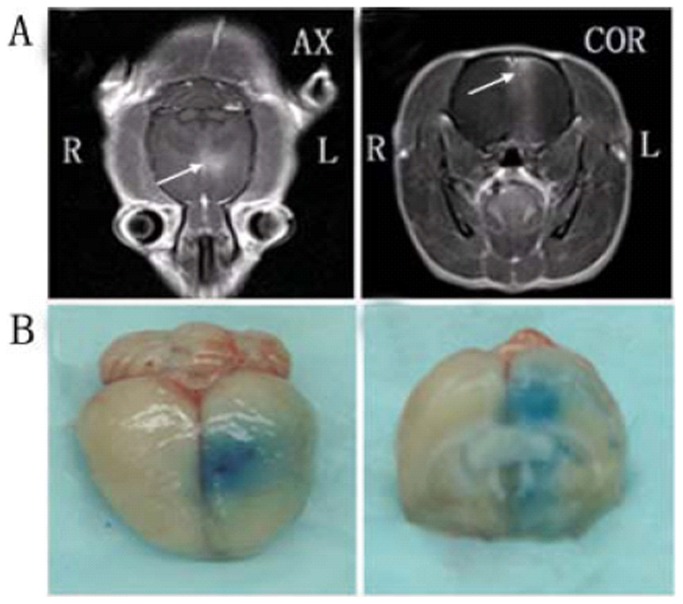
MRI monitoring of BBB disruption and photographs of harvested brain showing BBB disruption induced by focused ultrasound. (A) BBB opening was monitored by leakage of the MR contrast agent into the brain parenchyma on axial (AX) and coronal (COR) MR images (arrows). (B) The location of the BBB opening was confirmed by EB staining of the affected area.

BBB disruption was observed in the focal zone of the ultrasound beam with EB extravasation. The opening of the BBB by MRI-guided focused ultrasound was evaluated under optimum parameters according to the findings described above (ultrasonic conditions in Group 11). We monitored and confirmed BBB opening by MRI and leakage of the EB and MR contrast agent through the BBB into the cortex and caudate putamen of the brain after sonication. Leakage of the MR contrast agent to the brain parenchyma was observed on the MR images (Fig. 5A). The brain of each animal was harvested 4 h after sonication. The location of the BBB opening was confirmed by EB staining of the affected area (Fig. 5B).

### Localized Delivery of GDNF through the rat BBB and Monitoring MRI-guided Focused Ultrasound

**Figure 6:**
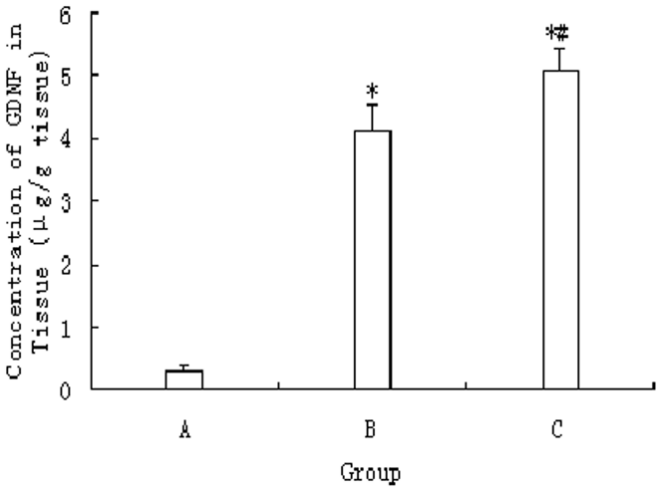
Concentration of GDNF in sonicated tissues after focused ultrasound-induced BBB disruption post-injection with GDNF (800 µg/kg) through the tail vein. (A) Control group (unsonicated group). (B) MB and GDNF group (GDNF group). (C) MB-GDNF group. *p<0.01, compared with Group A; ^#^ *p*<0.05, compared with group B.

The amount of GDNF delivered through the BBB using MRI-guided focused ultrasound was measured using an ELISA kit. The amount of GDNF in unsonicated tissues was 0.31±0.07 µg/g tissue, which was significantly lower than the values in the other groups (*p*<0.01). After sonication and a 0.5 ml bolus injection of MBs, the amount of GDNF in the target tissue increased to 4.12±0.41 and 5.07±0.37 µg/g, respectively. The GDNF concentration in tissue was significantly higher in the MB-GDNF group than in the GDNF group (*p*<0.05; Fig. 6).

## Discussion

Of the neurotrophic factors currently available, GDNF has proven to be remarkably effective in controlling PD and drug addiction [10], [18]. Brain-derived neurotrophic factor, another neurotrophic factor, has also been shown to be effective in treating depression and Alzheimer's disease [19], [20]. The therapeutic application of these neurotrophic factors and other therapeutic or diagnostic agents in the CNS is associated with a major and difficult problem: As the CNS is protected from exogenous substances by the BBB, GDNF, with a molecular size of 24 kDa, is easily blocked. The effective delivery of therapeutic agents into the brain can greatly improve the treatment of brain diseases and CNS disorders, such as neurological diseases, neurodegenerative diseases, and brain cancer [21], [22]. Various approaches have been used to open the BBB to facilitate drug delivery. For example, research has shown that the BBB could be opened by intra-arterial injection of a hyperosmotic solution, such as mannitol [23], [24]. However, this may cause penetration of non-targeted brain tissue and carries the risk of brain damage, bleeding, and infection. The application of focused ultrasound combined with MBs has shown potential in noninvasively delivering drugs across the BBB into targeted brain sites and that therapeutic agents can be delivered site specifically [7], [10].

Appropriate ultrasound exposure conditions are necessary when using focused ultrasound combined with MBs to open the BBB. The effects of exposure time, pressure amplitude, frequency, contrast agent type and dose, and repetition frequency on the magnitude of BBB disruption and histological effects on brain tissue have been investigated previously [11], [25], [26], [27], [28]. In the present study, the integrated effect of these parameters on BBB permeability was observed using EB extravasation. The 18 groups were divided according to an orthogonal design. The results showed that EB extravasation within the sonicated brain was higher in Groups 6, 11, 13, and 14 than in the other groups (Fig. 1). However, this does not mean that these conditions were optimal.

Further analysis was performed using histological examination. Erythrocyte exudation and a few apoptotic cells could be observed in the brain parenchyma in Groups 13 and 14 (Fig. 2). Endothelial cell damage showed that the BBB had been severely destroyed in Groups 13 and 14, as demonstrated by ultrastructural observation (Fig. 3). Although these pathological changes were not found in Groups 6 and 11, the number of apoptotic cells in Group 6 was significantly greater than that in Group 11 (Fig. 2B and D). The rate of apoptosis was significantly higher in Groups 6, 13, and 14 compared with Group 11, which can be ascribed to the ultrasound under different parameters. When the ultrasound was removed, the effect of the treatment on the cells disappeared and factor-reduced apoptosis was eliminated. The increasing trend of apoptosis did not appear with time (Fig. 2E). The EB extravasation results and histological examination indicated that the ultrasound exposure conditions in Group 11 (frequency, 1 MHz; MB dosage, 0.5 ml; exposure time, 1 min; pressure amplitude, 0.8 MPa; delay time, 60 s) were the optimum parameters. The subsequent experiments were performed under these conditions.

MRI has provided the necessary guidance for ultrasound-induced BBB disruption studies, including the placement of ultrasonic focus within the brain and assessment of BBB opening [28]. In the present study, MRI was used for real-time monitoring of the site of BBB opening induced by ultrasound combined with MBs. Signal intensity enhancement at the BBB disruption sites could be observed on the MR images, suggesting that BBB disruption could be predicted by the obtained MR images.

In some studies, therapeutic agents followed by MBs were injected intravenously. Another method of delivery is to attach the agents to the surface of MBs using a biotin–avidin bridging system [29] and then inject the combination intravenously. In our study, the adhesion efficiency of MBs was 63.1%±3.5%, which calculated the amount of loading GDNF on MBs. The bioactivity of GDNF bound on MBs was preserved throughout the process, as reflected by the in vitro differentiation of PC-12 cells (Fig. 4). The effects of the two methods mentioned above on GDNF delivery into the brain were then compared. The results showed that the GDNF concentration in the sonicated tissue after the injection of MB-GDNF was the highest (Fig. 6). MBs can be aggregated by ultrasound [30]. If GDNF is attached to the surface of MBs, then MB aggregation can increase the GDNF concentration in local tissue to promote GDNF delivery into brain tissue. The results of our previous study revealed that the MB combined with GDNF and the clinically diagnostic MB Sinovue did not differ in their effects on opening the BBB (data not shown).

In conclusion, our initial experiments suggest that 1 MHz frequency, 0.5 ml MB dosage, 1 min exposure time, 0.8 MPa pressure amplitude, and 60 s delay time are the optimum parameters for BBB opening induced by ultrasound combined with MBs. The MRI-guided focused ultrasound-induced BBB disruption method is a promising technique for the delivery of high-molecular-weight agents into the CNS. Under the aforementioned conditions, combining GDNF with MBs led to the greatest accumulation of GDNF in brain tissue, demonstrating that the biotin–avidin method is superior to other methods, such as direct injection. These suggest that MB-GDNF is a better way of delivering neurotrophic factors directly into the CNS and represents a novel effective method for the treatment of patients with CNS diseases. Specific brain regions were not discussed in this study, but GDNF permeability was detected under the optimum conditions. Analysis of specific brain areas in future research is thus warranted to verify the effects of ultrasound combined with MB-GDNF on the treatment of diseases.

## References

[1] Grondin R, Gash DM (1998) Glial cell line-derived neurotrophic factor (GDNF): a drug candidate for the treatment of Parkinson's disease. J Neurol 245: P35–42. 9808338 10.1007/pl00007744

[2] Airaksinen MS, Saarma M (2002) The GDNF family: signalling, biological functions and therapeutic value. Nat Rev Neurosci 3: 383–394. 11988777 10.1038/nrn812

[3] Carnicella S, Amamoto R, Ron D (2009) Excessive alcohol consumption is blocked by glial cell line-derived neurotrophic factor. Alcohol 43: 35–43. 19185208 10.1016/j.alcohol.2008.12.001 PMC2789656

[4] Volkow ND, Fowler JS, Wang GJ, Swanson JM, Telang F (2007) Dopamine in drug abuse and addiction: results of imaging studies and treatment implications. Arch Neurol 64: 1575–1579. 17998440 10.1001/archneur.64.11.1575

[5] He DY, Ron D (2006) Autoregulation of glial cell line-derived neurotrophic factor expression: implications for the long-lasting actions of the anti-addiction drug, Ibogaine. FASEB J 20: 2420–2422. 17023388 10.1096/fj.06-6394fje

[6] Nevalainen N, Chermenina M, Rehnmark A, Berglof E, Marschinke F, et al (2010) Glial cell line-derived neurotrophic factor is crucial for long-term maintenance of the nigrostriatal system. Neuroscience 171: 1357–1366. 20933580 10.1016/j.neuroscience.2010.10.010 PMC2991620

[7] Deng CX (2010) Targeted drug delivery across the blood-brain barrier using ultrasound technique. Ther Deliv 1: 819–848. 21785679 10.4155/tde.10.66 PMC3140134

[8] Pardridge WM (2007) Blood-brain barrier delivery. Drug Discov Today 12: 54–61. 17198973 10.1016/j.drudis.2006.10.013

[9] Tempany CM, Stewart EA, McDannold N, Quade BJ, Jolesz FA, et al (2003) MR imaging-guided focused ultrasound surgery of uterine leiomyomas: a feasibility study. Radiology 226: 897–905. 12616023 10.1148/radiol.2271020395

[10] Hynynen K, McDannold N, Sheikov NA, Jolesz FA, Vykhodtseva N (2005) Local and reversible blood-brain barrier disruption by noninvasive focused ultrasound at frequencies suitable for trans-skull sonications. Neuroimage 24: 12–20. 15588592 10.1016/j.neuroimage.2004.06.046

[11] Yang FY, Fu WM, Chen WS, Yeh WL, Lin WL (2008) Quantitative evaluation of the use of microbubbles with transcranial focused ultrasound on blood-brain-barrier disruption. Ultrason Sonochem 15: 636–643. 17910929 10.1016/j.ultsonch.2007.08.003

[12] Yang FY, Lin YS, Kang KH, Chao TK (2011) Reversible blood-brain barrier disruption by repeated transcranial focused ultrasound allows enhanced extravasation. J Control Release 150: 111–116. 21070825 10.1016/j.jconrel.2010.10.038

[13] Kaufmann BA, Lewis C, Xie A, Mirza-Mohd A, Lindner JR (2007) Detection of recent myocardial ischaemia by molecular imaging of P-selectin with targeted contrast echocardiography. Eur Heart J 28: 2011–2017. 17526905 10.1093/eurheartj/ehm176

[14] Kinoshita M, McDannold N, Jolesz FA, Hynynen K (2006) Targeted delivery of antibodies through the blood-brain barrier by MRI-guided focused ultrasound. Biochem Biophys Res Commun 340: 1085–1090. 16403441 10.1016/j.bbrc.2005.12.112

[15] Takalkar AM, Klibanov AL, Rychak JJ, Lindner JR, Ley K (2004) Binding and detachment dynamics of microbubbles targeted to P-selectin under controlled shear flow. J Control Release 96: 473–482. 15120903 10.1016/j.jconrel.2004.03.002

[16] Garbayo E, Ansorena E, Lanciego JL, Aymerich MS, Blanco-Prieto MJ (2007) Purification of bioactive glycosylated recombinant glial cell line-derived neurotrophic factor. Int J Pharm 344: 9–15. 17499462 10.1016/j.ijpharm.2007.04.003

[17] Gearhart DA, Middlemore ML, Terry AV (2006) ELISA methods to measure cholinergic markers and nerve growth factor receptors in cortex, hippocampus, prefrontal cortex, and basal forebrain from rat brain. J Neurosci Methods 150: 159–173. 16085318 10.1016/j.jneumeth.2005.06.009

[18] Carnicella S, Ron D (2009) GDNF–a potential target to treat addiction. Pharmacol Ther 122: 9–18. 19136027 10.1016/j.pharmthera.2008.12.001 PMC3682485

[19] Eisch AJ, Bolanos CA, de Wit J, Simonak RD, Pudiak CM, et al (2003) Brain-derived neurotrophic factor in the ventral midbrain-nucleus accumbens pathway: a role in depression. Biol Psychiatry 54: 994–1005. 14625141 10.1016/j.biopsych.2003.08.003

[20] Murer MG, Yan Q, Raisman-Vozari R (2001) Brain-derived neurotrophic factor in the control human brain, and in Alzheimer's disease and Parkinson's disease. Prog Neurobiol 63: 71–124. 11040419 10.1016/s0301-0082(00)00014-9

[21] Treat LH, McDannold N, Vykhodtseva N, Zhang Y, Tam K, et al (2007) Targeted delivery of doxorubicin to the rat brain at therapeutic levels using MRI-guided focused ultrasound. Int J Cancer 121: 901–907. 17437269 10.1002/ijc.22732

[22] Jordao JF, Ayala-Grosso CA, Markham K, Huang Y, Chopra R, et al (2010) Antibodies targeted to the brain with image-guided focused ultrasound reduces amyloid-beta plaque load in the TgCRND8 mouse model of Alzheimer's disease. PLoS ONE 5: e10549. 20485502 10.1371/journal.pone.0010549 PMC2868024

[23] Schwarze SR, Ho A, Vocero-Akbani A, Dowdy SF (1999) In vivo protein transduction: delivery of a biologically active protein into the mouse. Science 285: 1569–1572. 10477521 10.1126/science.285.5433.1569

[24] Doolittle ND, Miner ME, Hall WA, Siegal T, Jerome E, et al (2000) Safety and efficacy of a multicenter study using intraarterial chemotherapy in conjunction with osmotic opening of the blood-brain barrier for the treatment of patients with malignant brain tumors. Cancer 88: 637–647. 10649259 10.1002/(sici)1097-0142(20000201)88:3<637::aid-cncr22>3.0.co;2-y

[25] Chopra R, Vykhodtseva N, Hynynen K (2010) Influence of exposure time and pressure amplitude on blood-brain-barrier opening using transcranial ultrasound exposures. ACS Chem Neurosci 1: 391–398. 20563295 10.1021/cn9000445 PMC2885821

[26] Wang F, Cheng Y, Mei J, Song Y, Yang YQ, et al (2009) Focused ultrasound microbubble destruction-mediated changes in blood-brain barrier permeability assessed by contrast-enhanced magnetic resonance imaging. J Ultrasound Med 28: 1501–1509. 19854965 10.7863/jum.2009.28.11.1501

[27] McDannold N, Vykhodtseva N, Hynynen K (2008) Effects of acoustic parameters and ultrasound contrast agent dose on focused-ultrasound induced blood-brain barrier disruption. Ultrasound Med Biol 34: 930–937. 18294757 10.1016/j.ultrasmedbio.2007.11.009 PMC2459318

[28] Kinoshita M, McDannold N, Jolesz FA, Hynynen K (2006) Noninvasive localized delivery of Herceptin to the mouse brain by MRI-guided focused ultrasound-induced blood-brain barrier disruption. Proc Natl Acad Sci U S A 103: 11719–11723. 16868082 10.1073/pnas.0604318103 PMC1544236

[29] Hernot S, Klibanov AL (2008) Microbubbles in ultrasound-triggered drug and gene delivery. Adv Drug Deliv Rev 60: 1153–1166. 18486268 10.1016/j.addr.2008.03.005 PMC2720159

[30] Lum AF, Borden MA, Dayton PA, Kruse DE, Simon SI, et al (2006) Ultrasound radiation force enables targeted deposition of model drug carriers loaded on microbubbles. J Control Release 111: 128–134. 16380187 10.1016/j.jconrel.2005.11.006 PMC1526414

